# Multidisciplinary analyses on the 11^th^-12^th^ century bronze doors of San Marco, Venice

**DOI:** 10.1371/journal.pone.0288094

**Published:** 2023-07-13

**Authors:** Marianne Mödlinger, Mauro Bernabei, Jarno Bontadi, Marco Fellin, Martin Fera, Giorgia Ghiara, Martino Negri, Judith Utz

**Affiliations:** 1 IMAREAL, Paris Lodron University Salzburg, Krems/Donau, Austria; 2 CNR-IBE, Consiglio Nazionale delle Ricerche, Istituto per la Bioeconomia, San Michele all’Adige, Italy; 3 Institut für Urgeschichte und Historische Archäologie, Universität Wien, Vienna, Austria; 4 Department of Environmental Science and Policy (ESP), Universitá degli Studi di Milano, Milano, Italy; VIT University, INDIA

## Abstract

Two 11th- and 12th-century entrance doors from the Basilica di San Marco in Venice, made of different copper alloys and woods, were non-invasively examined in situ. The chemical composition of the metals, the way in which different metal parts were joined together, the tree species used to construct the supporting structures and the age of the wood are determined. A portable ED-XRF instrument and optical microscopes were used. The doors were also photographed to produce high-resolution orthophotos and 3D models. The metal parts of the doors were made of leaded tin-bronze and quaternary Cu-Sn-Zn-Pb alloys and were mounted on a wooden multi-layer structure of larch and silver fir; the dendrochronological dates of some of the larch boards are 1965, *teminus post quem*.

## 1. Introduction

Doors were and still are mostly made of wood, but in certain cases they were reinforced and embellished with copper alloys, such as the gates of temples or palaces. This tradition was consciously continued in the Middle Ages, especially by the Church. Here, the doors, whose bronze surfaces were usually decorated with images of biblical scenes, still had a liturgical-ritual significance, as they were also considered to be the gates to paradise. The bronze doors that survive today represent the only surviving complex of large medieval bronzes in Europe, most of which date from the 11^th^-12^th^ century. Today, about 30 bronze doors from this period have survived, most of them in Italy, three in Germany and one each in Poland and Russia.

The most common feature of these doors is their metal parts, made of different copper alloys. The metal parts were made by the lost wax process; they could be cast as a whole, to get a self-supporting structure, or individual metal fittings were attached to a wooden support. During the last centuries, many wooden supporting structures have undergone restoration and replacements which often have resulted in a rearrangement of the bronze plates of the doors: the current order of them thus does not necessarily correspond to the original one. Some doors have inlays of various materials that allow a coloured depiction of different biblical scenes or saints.

**Fig 1:**
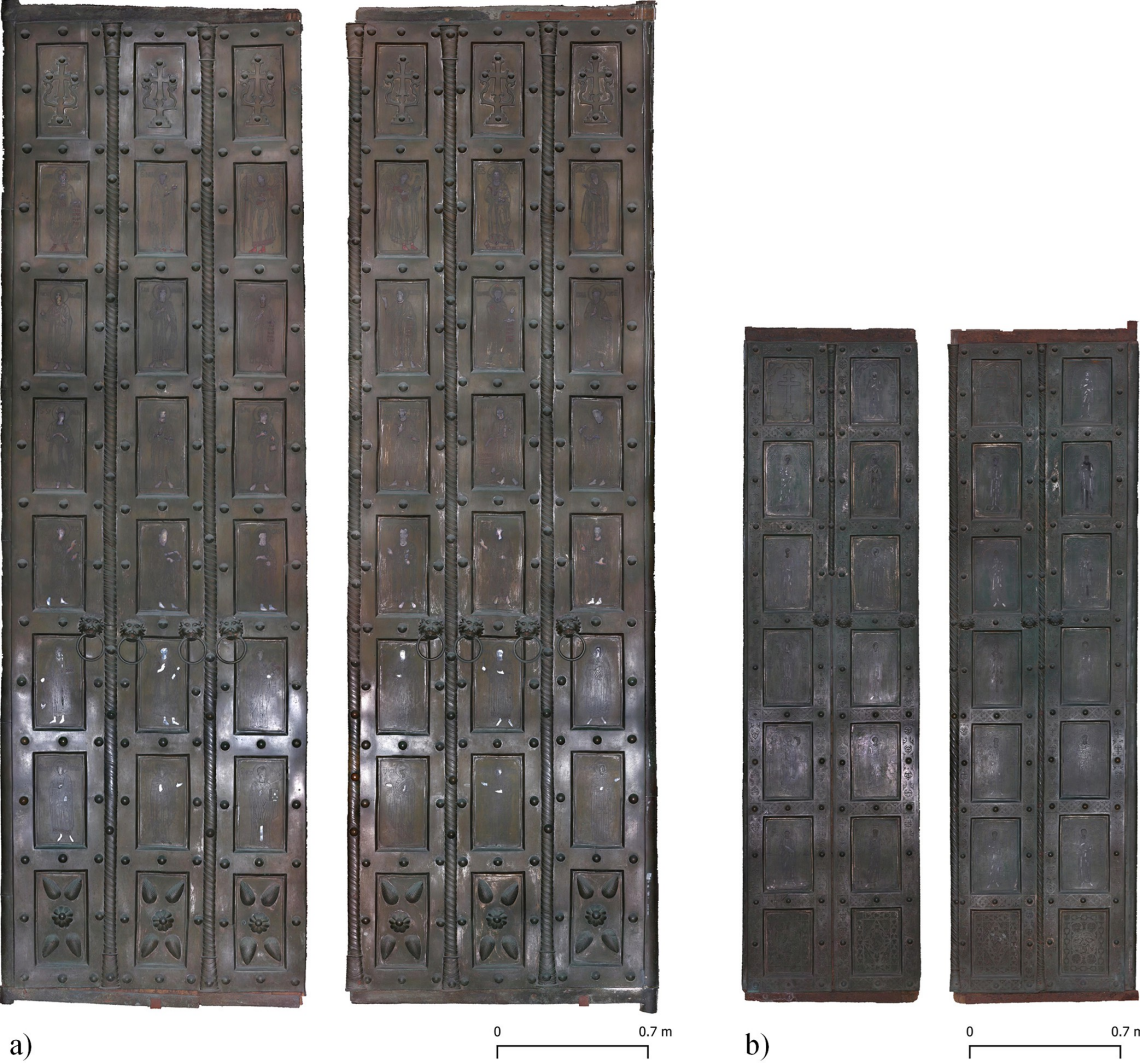
Basilica di San Marco, Venice, Italy. Orthophotos of the two 11^th^–12^th^ century bronze doors studied. Left: main door; right: door San Clemente. The shiny areas in the lower part of the doors are those where people touch the doors most.

**Fig 2:**
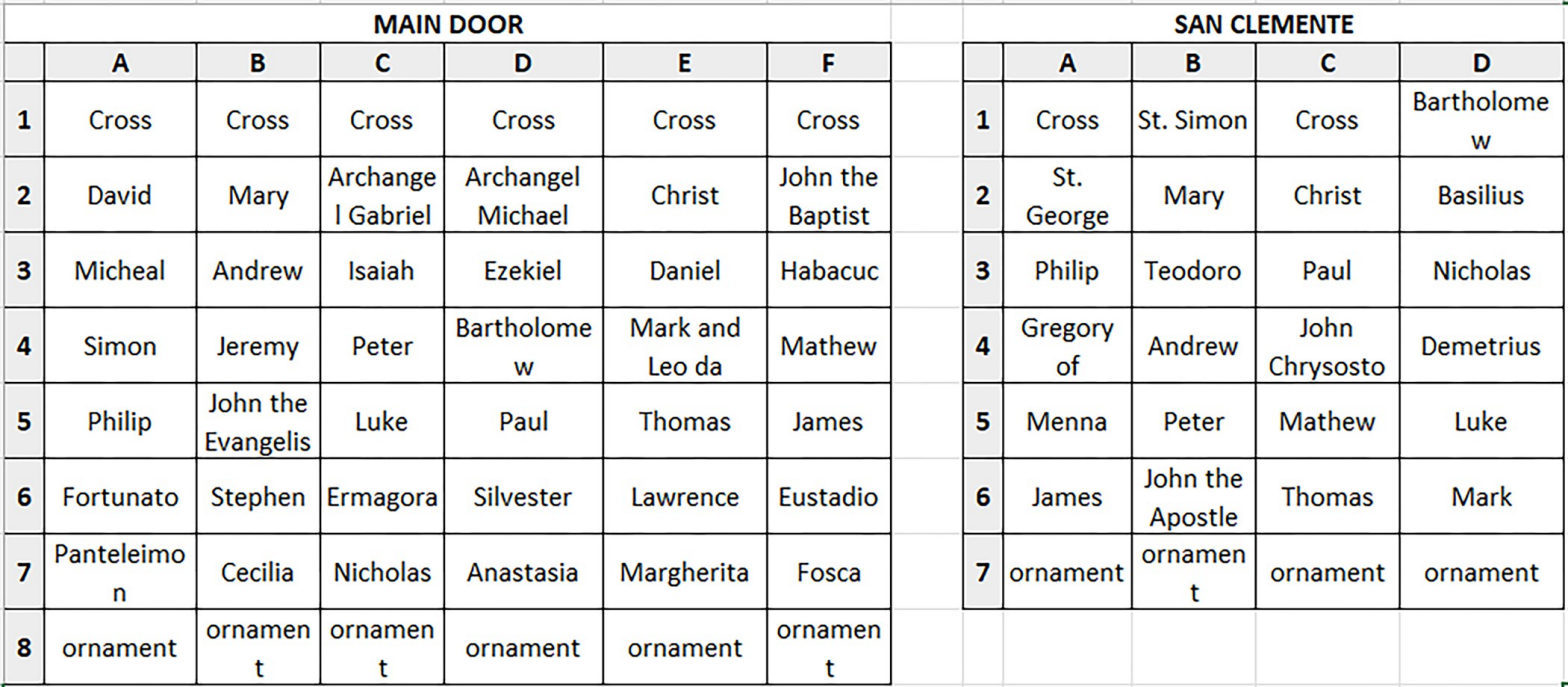
Basilica di San Marco, Venice, Italy. Plate overview and numeration of both bronze doors. Left: main door; right: door San Clemente.

Like other bronze doors, the two doors from the Basilica di San Marco in Venice (**Figs 1** and **2**), which are the focus of this paper, have inlays of various materials that allow for multi-coloured depictions on flat plates of copper alloy. Only the upper and lower registers show ornamental motifs in bronze relief. The pictorial programme of both doors consists of standing figures of Christ, Mary, the Apostles, the Evangelists, the Fathers of the Church and proto-martyrs (in the case of the main door, prophets and archangels are also added). Both doors belong to the so-called "Byzantine Doors", a group of eight doors in Italy that were probably made (partially or completely) in Byzantium (today’s Istanbul, Turkey) in the second half of 11^th^ century and the first half of the 12^th^ century.

Among the eight surviving Byzantine doors, the two Venetian ones occupy a special position because of their unique characteristics. Neither inscriptions on the objects themselves—as in the case of the doors of Monte Sant’Angelo [1], San Paolo fuori le Mura, Salerno and Atrani—nor contemporary references in texts—as in the Chronicon of Montecassino, which deals with the doors of Amalfi and Montecassino [2, 3]—could shed any light on the circumstances of their construction. The larger of the two doors studied, located at the main portal of the basilica, depicts and names Leo da Molino, ’procuratore’ of San Marco between 1112 and 1138 [2, 4], in proskynesis before St Mark, thus giving a clue to the donor and the date of the door. Instead, since the late 18^th^ century, it has been assumed that the smaller door of San Clemente, located on the south of the main portal, was a gift from the Emperor Alexios Komnenos (1081–1118 CE) to the Venetians. This has been largely handed down by later art historical research, but reliable sources are lacking [3–6]. Since this door resembles the door of San Paolo fuori le Mura in Rome (dated 1070 CE), both in its construction and in the ornamental design of the frames, it was assumed that the Porta San Clemente was also made in Constantinople in this period.

The two doors in Venice are very different in construction and decoration. The frames of the smaller door are completely decorated with engraved floral ornaments and the figures on the plates stand in front of an arcade, while the main door lacks these features. Consequently, based on stylistic features, some authors suggest different places of production for the two Venetian doors: possibly the main door was made locally as a copy of the smaller one [2, 7]. The Byzantine doors of southern Italy all date from the second half of the 11^th^ century, while the main door of San Marco seems to have been made between 1112 and 1138 CE. It is possible that the former Constantinopolitan workshop no longer existed and that other artists, perhaps less trained in the decoration of bronze/brass plates, were responsible for the production.

Previous studies have paid little attention to the production process of bronze doors, the choice of materials and the interaction between the material and the images. Only one of these bronze doors, the door from Monte Sant’Angelo—Italy, has recently been studied in detail. The analyses presented here are part of an Austrian project investigating 1) the materials and techniques used to create the doors; 2) how and which craftsmen, artists and patrons were involved in the production process; and 3) how the materials used and the pictorial representations on the doors together create meaning. With the help of the analyses presented here, we hope to shed light on some of the still unclear aspects of the bronze doors of the Basilica di San Marco, Venice.

## 2. Methodology

### 2.1 Materials

This study focuses on the two 11^th^ and 12^th^-century metal doors of the Basilica of San Marco in Venice. Both doors are located on the west façade of the basilica: the central main door (hereafter MD) and the door of San Clemente (SC), south of the main door. Both doors consist of a wooden base on which the metal plates and the frames, which cover the gaps between the plates, are mounted. All frames were brazed together; also the frames of a column of plates were brazed together. The gaps between the individual brazed "frame columns" are covered by cast copper alloy spiral bars (main door: between columns AB, BC and DE, EF plus an additional spiral bar to cover the gap between the two door leaves, i.e. between columns CD; San Clemente: between columns AB and CD and between the two leaves, i.e. between columns BC).

As in the case of the other Byzantine doors, the elementary pieces that make up the frames of the two Venetian doors are perfectly soldered together. The surfaces were then smoothed. As a result, it is almost impossible to determine the exact size of each piece without an X-ray examination (**Fig 1**). Chemical analysis, however, makes it possible to reconstruct the shape of the frame elements with a good approximation. As in the case of the main door, the top and bottom pieces are mostly cast in "L" or "U" shapes, while in the centre of the door they are mostly "├" or "H" shaped. Small cracks or indentations on the inside, i.e. the edge around the panels, provide clues as to where the pieces join (**Fig 1**). In the San Clemente door, the frames are also made of different pieces that are soldered or mechanically joined; "U" and "H" shapes are likely because each column of panels is separate from the others, and some frames have visible joints (**S1 Fig**).

### 2.2 Image-based modelling

Detailed photographic documentation of the doors was undertaken to document and support further analysis. The primary objective was to create a metric basis for mapping the exact measurement points, which would reflect both the geometry and texture of the surface in high resolution. In addition to regular high-resolution photography of individual plates, both doors were recorded using image-based modelling.

Image-based modelling is a method based on photogrammetry that uses a series of 2D photographs taken from different angles to reconstruct surfaces in three dimensions [8]. It’s use in archaeology and art history has been steadily increasing in recent years, as it allows the creation of highly detailed and accurate digital replicas of cultural heritage objects, architecture and other artefacts. This is particularly useful for the conservation and documentation of fragile or inaccessible objects, and for the creation of virtual exhibitions and educational materials [9, 10].

The spatial situation at the site of the doors in Venice only allowed the recording of individual door leaves in the open position as single parts. Data acquisition was carried out in parallel with the chemical and dendrochronological analyses. The images were taken with a Ricoh GR IIIx camera with a focal length of 26.1 mm (57° diagonal) and an RGB primary colour CMOS sensor (23.5 mm x 15.6 mm, 24.24 megapixels, pixel pitch 3.9 μm) mounted on a 3.5 m long hand-held carbon rod. Illumination was provided by an array of three manual indirect flashes mounted on a rig with a reflective umbrella. Agisoft Metashape software (version 1.7.2) was used for processing. The scale was determined by measuring different distances on the object, which were used as scale bars during processing.

**Fig 3:**
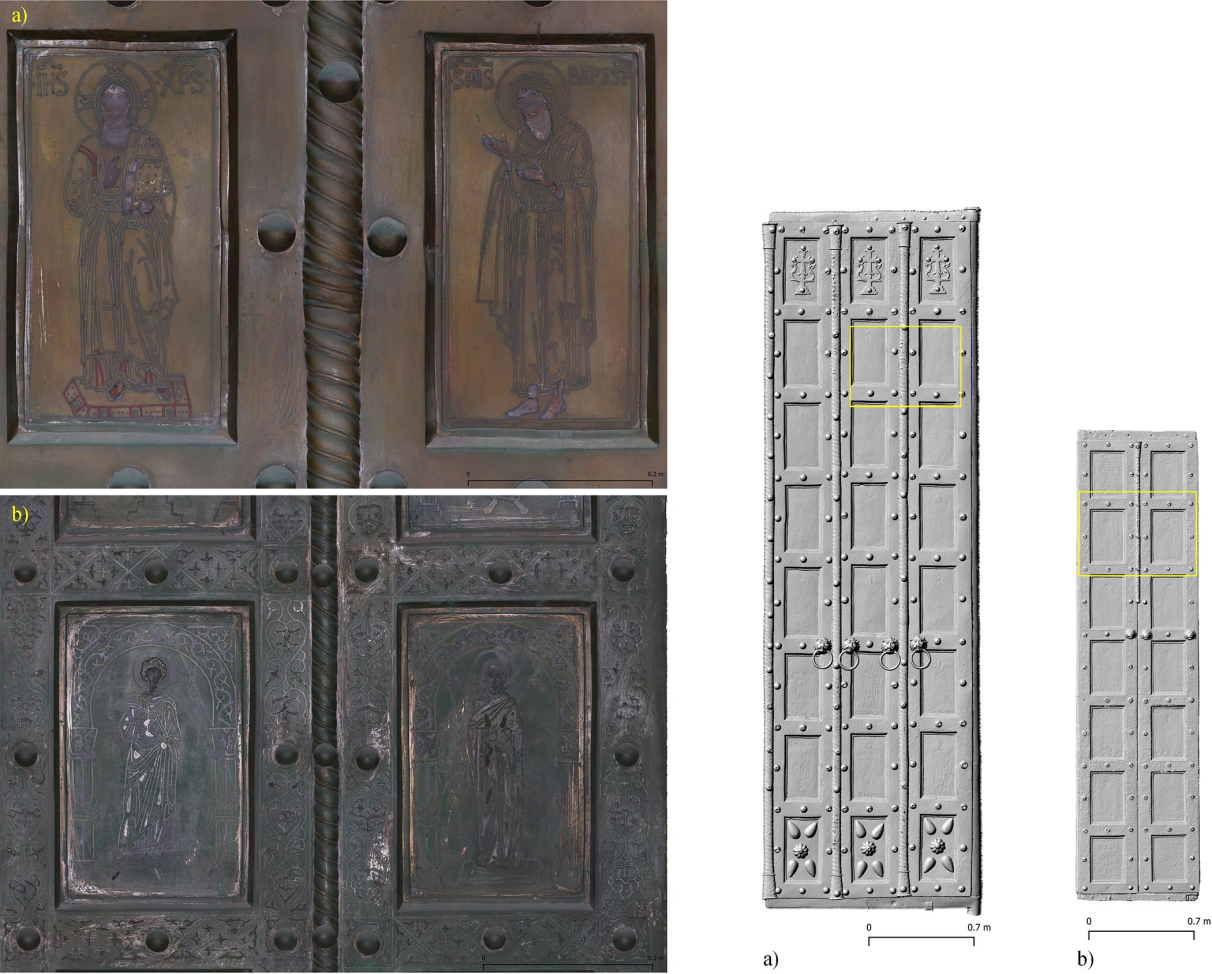
Basilica di San Marco, Venice, Italy. Orthophoto detail, shaded Digital Surface Model. **a)** Main door, right leaf, plates E2 (left) and F2 (right). **b)** Door San Clemente, left leaf, plates A2 and B2.

As an example, the right leaf of the main door (max. extent 4.7x1.45 m, coverage area 6.63 m^2^) was covered by 444 images at an average perpendicular distance of 1 m. The overlap is over 60% and most parts are covered by at least 6 images. The resulting Digital Surface Model (DSM) has an object resolution of 0.141 mm/pixel. On this basis a True Orthophoto was generated as an orthomosaic with a resolution of 0.2 mm/pixel using an orthographic projection normal to the surface plane (**Fig 3**).

Further processing included the generation of a textured 3D model based on a derived point cloud of 30 million points. Due to the spatial situation, a separate model was generated for each door leaf, while the back of the SC was also modelled in a closed state. For the MD only a photographic documentation of the backside was carried out due to the limited space on site.

### 2.3 Chemical analyses and Principal Component Analysis (PCA)

Chemical analyses were carried out using an Oxford Instruments portable ED-XRF analyser, model X-MET5100, equipped with a high-resolution detector 45 kV Rh target X-ray tube (max 50uA). The X-ray beam allows spot measurements of approximately 9 mm in diameter. The main elements such as Cu, Sn, Zn and Pb were detected quantitatively, other elements such as Ag, Pd, As and Fe only qualitatively, mainly due to the influence of corroded layers. S is not detectable by this ED-XRF. Measurements were made using the standard setup of 40 kV voltage, 10 μA current and 60 s acquisition time. Alloys as indicated in **S1 Table** were used as calibration standards.

**Fig 4:**
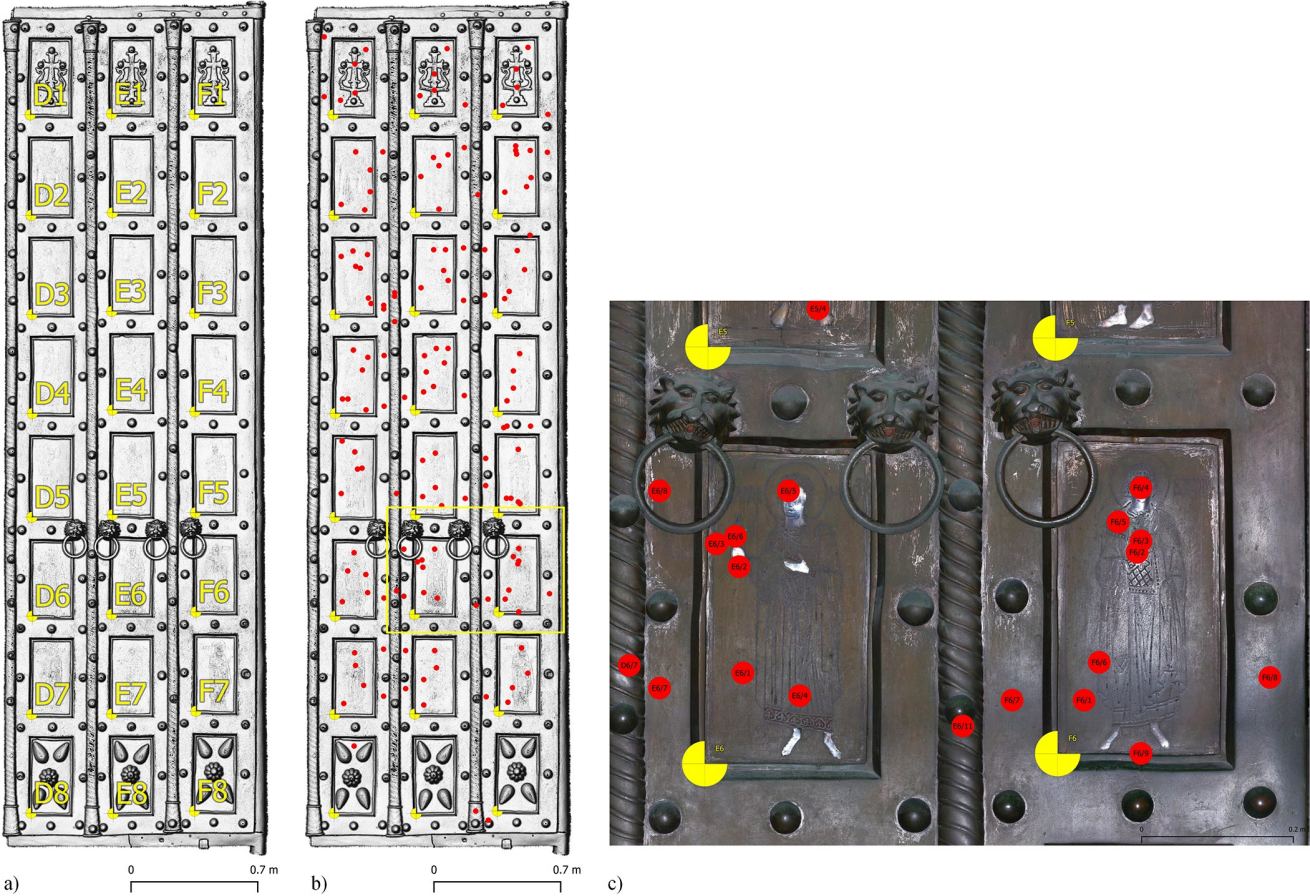
Basilica di San Marco, Venice, Italy. Digital Surface Model visualisation and detail of the orthophoto. **a)** Main door, right leaf, plates mapping. **b)** main door, right leaf, mapping of ED-XRF measurement points. **c)** Main door, right leaf, mapping of ED-XRF measurement points mapped on orthophoto (plates E6 and F6), as indicated in b).

On each plate of both doors, between 4 and 10 analyses were taken on the metal part (**Fig 4**). The mean of each piece was used for data processing. Additional analyses were carried out on the frames, columns, hinges and lion heads. Inlays were also analysed on both doors; however, as the inlay is usually thinner and smaller than the measurement area, quantitative results could not be obtained as the background composition of the plate was always measured together with the inlay. Further non-invasive analyses (micro-XRF mapping, SWIR imaging, RAMAN and FTIR analyses) are planned for the summer of 2023.

Copper alloy surfaces exposed to the elements degrade and corrode over time. These processes are usually associated with the depletion of copper and the formation of various corrosion products, as well as the accumulation of dust and dirt on the surface of the object. Brushing the surface of the measurement areas removes significant amounts of dust and dirt, but corrosion products remain. Consequently, surface measurements must be considered as qualitative, especially in terms of minor and trace elements. In order to assess and reduce the influence of corrosion on the analyses, multiple measurements were taken on each metal part. As the doors were restored at the end of the 1960s and any corrosion product was removed during the restoration, they have corroded quite uniformly over the last 50 years; only a very thin surface corrosion layer was found, whose influence on the main alloying elements Cu, Sn, Zn and Pb was considered to be redundant, so the results for the alloying elements were considered to be quantitative.

Due to the large amount of chemical data and variables, a chemometric approach was followed. It was helpful to highlight: i) the correlations between the variables and the relative importance of each of them within a given system; i) the relationships between the samples. A Principal Component Analysis (PCA) was therefore performed on the data matrix. This procedure aims to extract the maximum possible information contained in a multivariate data structure by translating it into linear combinations of the variables [11].

From a geometric interpretation, PCA consists of a process of rotation of the original data set into a new geometric space: the x-axis of the new geometric plane (which will constitute the principal component one or PC1) is oriented in the direction of the maximum variance of the data, and the y-axis (principal component two or PC2) is perpendicular to the first (in the direction of the next maximum variance of the data). The variance (σ2) is defined as the dispersion of the numbers in a data set with respect to the mean ($\bar{X}$) (Eq 1) [12]:

$$\sigma^{2}=\sum_{i=1}^{n}(x_{i}-\bar{X})/n-1$$ (1)

where *x_i_* is the value of the i^th^ element (term in the dataset) and n is the sample size (number of samples).

The number of these new axes (PCs) is equal to the number of the original variables. Their directions with respect to those of the original axes (the original variables) are determined by the eigenvectors, vectors of the new space, expressed by coefficients (loadings) in the range ± 1 [13]. Each eigenvector is associated with an *eigenvalue* m representing its explained variance: in general, higher eigenvalues are associated with relevant information (useful, modelling, interpretable, etc.), while lower eigenvalues are associated with low variability due to noise or irrelevant information [12].

The PCA thus produces two graphs corresponding to: i) loadings; ii) scores. The loadings are defined by a matrix in which the columns represent the *eigenvectors* and the rows represent the original variables. Each row contains the numerical coefficients that define the importance of each original variable in that eigenvector. The scores are the projections of the samples in the newly defined space and are defined by a matrix where the rows are the samples and the columns are the principal components. They therefore appear as the data in the new geometrical space (the PC space) [12]. The value of the scores is the result of a linear combination in which the variables are the original variables (usually centred and scaled) and whose multiplicative coefficients are the loadings of each component (Eq 2).

$$S_{O,P}=X_{O,V}L_{V,P}$$ (2)

where S equals the scores, X is the original matrix and L represents the loadings [14]. A biplot can also be used, which is a combination of loadings and scores in the new geometric space. The calculation was performed with CAT (Chemometric Agile Tool) software [15] on the raw data, centred and autoscaled.

### 2.4 Wood analyses

Analyses on the wooden structures of both doors aimed of identifying the tree species used and dating any elements that met the requirements for dendrochronological dating, considering that nothing could be dismantle or invasively sampled. The Venetian doors both show a multi-layered structure of wood supporting the bronze elements. From the backside of the doors there is a first layer of wooden planks laid horizontally and then, under this first, a second layer of planks laid vertically. The wooden planks of both doors are painted with a thick green paint, which makes it possible to see the wooden structures only where the paint has been worn away. On both leaves of the San Clemente door, it was possible to observe the cross section of the vertical boards on their upper sides. Conversely, on the main door, all the cross sections of the boards were obscured by paint or metal structures, making any attempt to observe the width of the annual rings futile.

**Fig 5:**
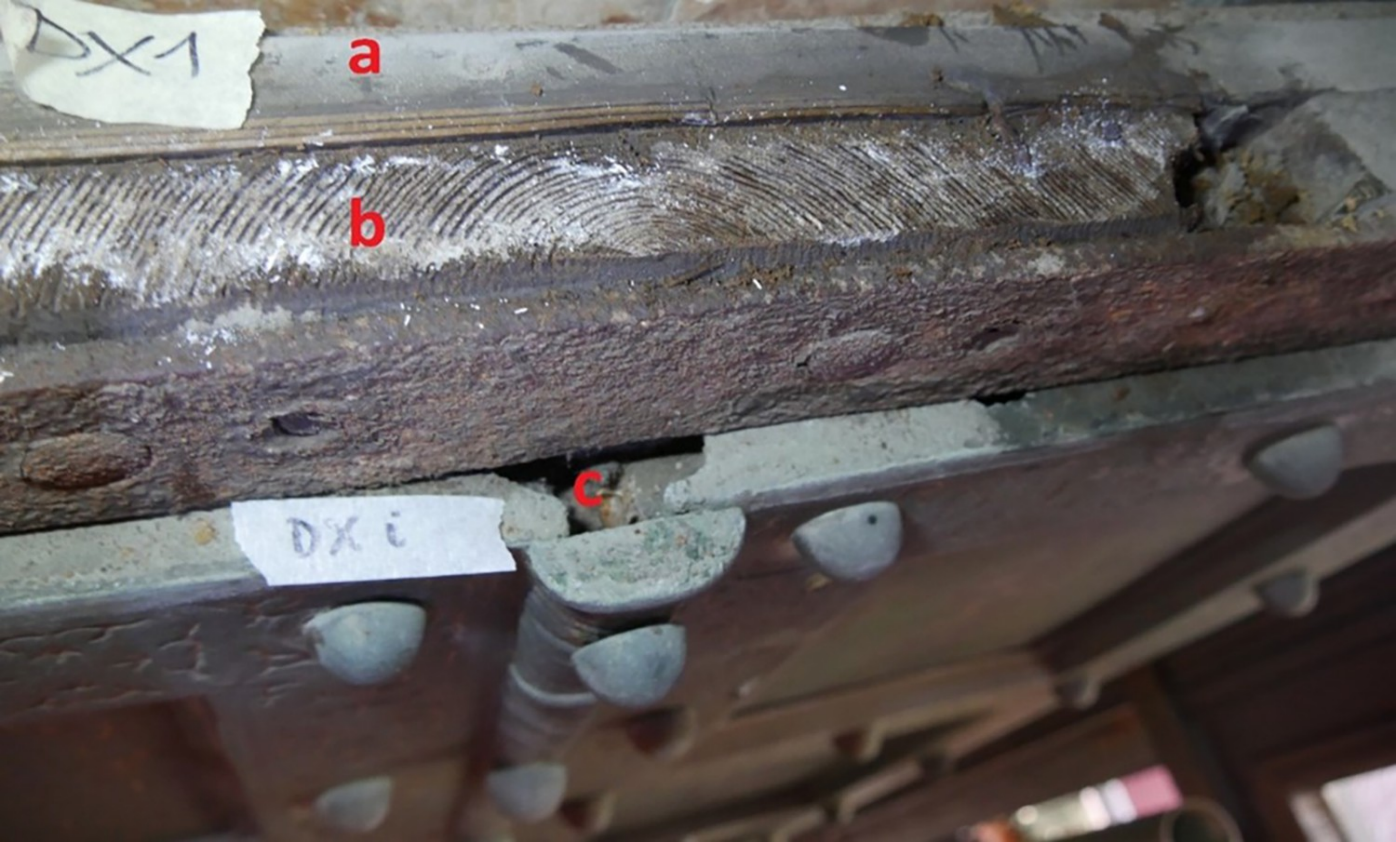
The upper side of the right leaf of the San Clemente door. **a)** The first layer of horizontal planks. **b)** The second layer of planks placed vertically: the annual rings on the cross section are highlighted with chalk powder. **c)** The gap through which a third layer of wood could be glimpsed.

Investigations on the upper sides of the San Clemente door revealed a third layer of wood seen through the gaps in the metal cover (**Fig 5**). This layer appears to be in direct contact with the bronze plates and, as far as can be determined by examining it through thin gaps, it would appear to be older than the other two layers of boards. Using tweezers and a scalpel, small samples were taken from both doors to verify the tree species, while, on San Clemente door, photographs were taken on the cross sections of the vertical planks for wood dating. Cross sections are the most useful surface for accurately measuring the annual ring widths used in dendrochronological dating.

Dendrochronology is the study of tree rings in relation to time. It is based on the principle that the width of annual rings is largely determined by the conditions under which the plant grew. Tree rings are dated by comparing the ring sequence of a sample with a corresponding, previously dated reference chronology, which should be of the same tree species, from approximately the same geographical area as the wood being dated, and obviously long enough to cover the prospective time period covered by the wood sample. As stated in the reference standard [16], the dendrochronological date obtained refers to the outermost (i.e. most recent) annual ring visible on the artefact, which does not necessarily represent the year of manufacture of the object.

A total of 7 small wood samples were taken from non-visible parts of both doors, 2 from the main door and 5 from the San Clemente door (**Table 1**). The identification of the tree species was carried out by observing the microscopic features of the samples with the Olympus CX41 transmitted light microscope, according to the reference standard UNI 11118, 2004 [17], and by comparison with the descriptions given in the main wood anatomy atlases [18–21].

**Table 1:** Samples collected on venetian doors. WE: waney edge, the last growth ring, close to the bark, produced by the tree before falling. SW: sapwood, the external part of the trunk that in larch, as in many species, differs in colour from the inner portion.

Sample	Door	Leaf	Description	Species	Date	WE	SW
1	Main door	left	First layer of horizontal boards	Larch	-	No	No
2	Main door	left	Second layer of vertical boards	Larch	-	No	No
3	San Clemente	right	Second layer of vertical boards	Larch	1695	No	No
4	San Clemente	left	Second layer of vertical boards	Larch	1695	No	No
5	San Clemente	left	Second layer of vertical boards	Larch	1695	No	No
6	San Clemente	left	Third layer board next to bronzes	Silver fir	-	No	-
7	San Clemente	right	Third layer board next to bronzes	Silver fir	-	No	-

Tree-ring widths for dendrochronological dating were measured directly on the photographs taken on the upper side of San Clemente door with a LINTAB (LINear TABle, Rinn Thech, Germany) and recorded using the TSAP-Win software (Rinn Tech, Germany). Analysis taken into account the wooden elements showing a sufficient number of annual rings. The ring curves of the latter was then visually and statistically cross-matched with the individual ring curves of the other San Clemente door elements in order to ensure measurement accuracy and to detect the presence of xylological anomalies, such as false or missing rings. Having shown a reciprocal matching, the individual tree-ring series were then averaged to produce a representative tree-ring mean series of the door. PAST4 and TSAP-Win software were used to visualise the series and for carrying out statistical tests.

## 3. Results and discussion

Previous studies carried out during and after the last restoration of the doors at the end of the 1960s [6] identified on the surfaces of both doors: dust, organic substances such as proteins and fats, and a total encrustation of microcrystals of sulphates, copper chloride carbonates, copper oxides and sulphides, and oxides of silver and lead. The metal parts of the doors then underwent restoration treatments such as: washing with various solvents (not specified) to dissolve the grease and facilitate the removal of the dust trapped in the interstices; electrolytic treatment with titanium electrodes and deep electric brushing of the surfaces and interstices until the corrosion layers were removed from the metal surface. Unfortunately, the corrosion process started again because the previous protective layers were also removed. Today, the doors are in a very poor state of repair and have been severely corroded again in less than 60 years.

### 3.1 Image-based modelling

**Fig 6:**
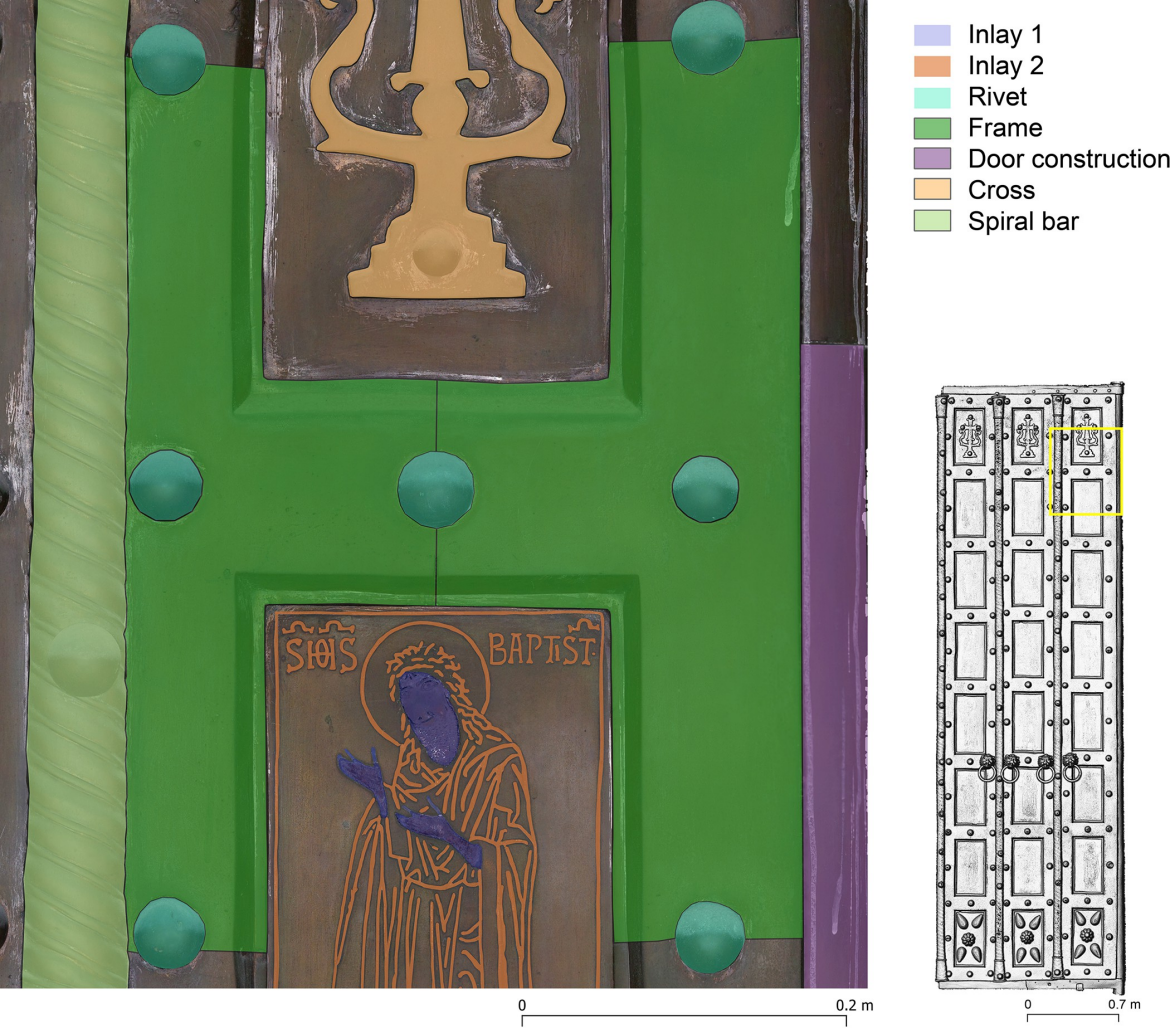
Basilica di San Marco, Venice, Italy. Main door, right leaf, plates F1 (top) and F2 (bottom). Background Orthophoto/DSM, construction elements and chemically analysed entities.

The use of image-based modelling within the project has proved to be of great benefit on many levels. It supports the spatial documentation of the chemical analyses, but also helps in the interpretation of the construction and shows the current conservation status of the investigated objects. The collected data was accumulated in a desktop GIS environment, where tabular data from chemical analyses, geometric data from 3D modelling and spectral data from photographs were used to organise and visualise the results (**Fig 6**). In addition, the high spatial resolution of the orthophotos and DSMs collected during the project will allow comparative studies beyond a single door to examine details of production processes and connections that indicate specific workshops.

For long-term preservation, the data will be prepared for the IMAREAL repository at the University of Salzburg. Semantic annotations based on the ontology of the well-established RealOnline database will be added. This will support and enable future research on this corpus by making it more accessible to a wider audience. It can foster collaborative research and provide a tool for the digital preservation of these artworks by forming the basis for the development of Heritage Digital Twins, a concept that has been introduced in the field in recent years [22].

### 3.2 Chemical analyses and PCA

#### 3.2.1 Inlays

**Fig 7:**
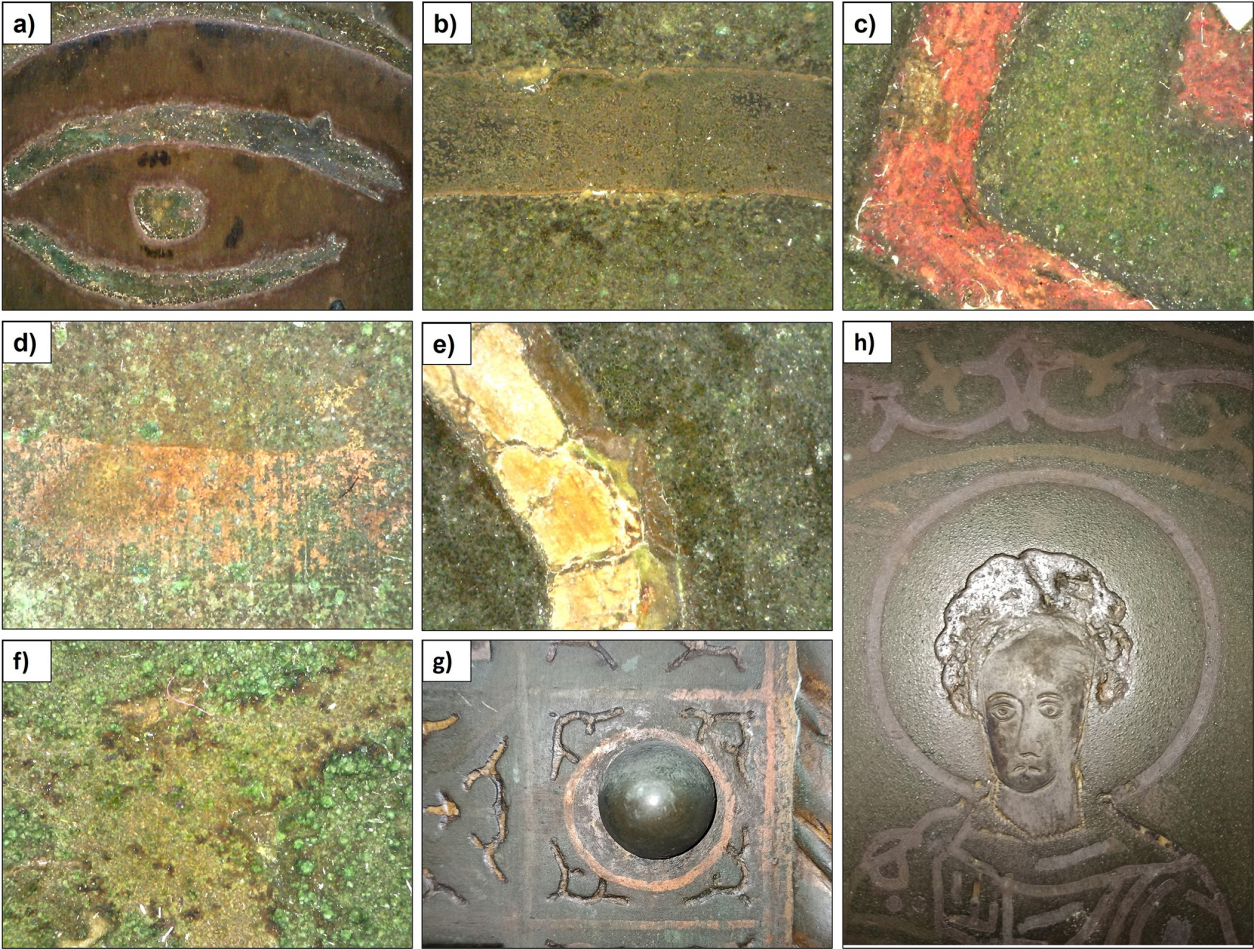
Details of some of the inlays documented. **a)** silver (MD; plate B3, eye of the saint). **b)** niello (MD; plate B3). **c)** red (cinnabar; MD, plate D3). **d)** copper (SC; frame between plates C7 and D7). **e)** yellow (SC; frame left of plate C5). **f)** surface treatment (?) as decorative element (SC; plate B4) **g)** copper and yellow (SC; frame between plates C7 and D7). **h)** San Filippo (SC, plate A3), showing silver plating (face), niello inlay and surface treatment (?) as decorative elements.

Both bronze doors contain inlays of different colour and chemical composition. Quantitative results were not obtained with ED-XRF, firstly because S, a major compound of niello and cinnabar, cannot be detected with ED-XRF, and secondly because the inlay is too thin to be measured with ED-XRF alone; the chemical composition of the plate is always part of the result. Nevertheless, the following inlays have been identified (**Fig 7**):

1. Silver (Ag): the face, the hand and most of the feet of the person portrayed, as well as parts of their clothing;
2. Red (cinnabar; main elements detected: Cu, Hg): clothes, shoes, accessories (only on MD);
3. Reddish (Cu): decorative elements (only on SC);
4. Yellow (unknown): decorative elements on the plate and the main bronze structure of the side door (only on SC);
5. Gold (Au): on silver parts, associated with gilding.

On the main door, inlays were found only on the plates; the frame itself is undecorated. Silver has been found on the face, the hands and most of the feet of the person depicted, as well as on parts of his or her clothing. It was applied by plating, i.e. hammering thin sheets of silver into the cavities for the head, hands and feet in the plates. These cavities were chiselled out of the flattened and polished plate to ensure a firm hold for the silver plating. The silver plating sometimes contained up to 2% Au by weight, which may have been associated with earlier gilding. Niello (a black mixture of sulphur, copper, silver and lead) was used mainly for the depiction of clothing and the surrounding decoration of the saints. It was added as a paste or powder, heated until molten/softened, and then pressed into the engraved lines in the plate. As it is impossible to measure niello alone without the background, we have not been able to determine whether silver sulphide niello was used or not. Red colours (cinnabar or vermilion; HgS) were used for the saints’ clothes, shoes and various accessories, such as books. Cinnabar was also used for some decorative elements. In addition to Hg, Re and sometimes Au were also found, up to 3.1 and 1.9 wt.% respectively. Traces of Se and Br were also found.

Like the main door, the side door of San Clemente also has inlaid work in a variety of colours. In addition to silver plating and inlays, niello inlays were also noted. Both the silvering and the inlays contained up to 3.5% Au by weight, consistent with the MD decoration. In contrast to the main door, no traces of red decoration (cinnabar) were found. The frames of the San Clemente door are also richly decorated. They contain inlays that are not present on the plates (except for plates A1 and C1) or on the main door. These inlays are made either of copper or of a yellowish compound that has not yet been identified. The copper inlays are mainly found as straight lines or circles around the buttons of the frame. The other type of inlay has a yellowish colour, is very brittle and has an as yet unidentified chemical composition. Finally, a special surface treatment allowed the appearance of more decorative elements (**Fig 7F** and **7H**)–these are certainly not inlays.

#### 3.2.2 Plates, frames and decorative elements

The PCA was performed on the ED-XRF data of the two door frames (F), plates (P) and decorative elements, namely lion heads (L) and spiral bars (B), in a matrix of 286 rows (the ED-XRF analysis sites) and seven columns (alloy elements). The raw data provided information on the correlations between variables (alloy constituents) and the distribution (patterns/trends/clusters) of the samples (analysis sites). The ED-XRF measurements were labelled according to **Fig 2** and numbered sequentially (**S2 Table**). The first PCs (PC1, PC2 and PC3) carry the most information about the dataset with explained variance of 37.5%, 20.3% and 18.8% respectively. The loadings in the biplot (variable labels highlighted in pink) clearly show the negative/positive correlations between the elements of the alloy.

The plot appears as a 2D graph where the x and y axes represent the variance of each variable explained by PC1 (37.5% of the variance) and PC2 (20.3% of the variance). The alloying elements are distributed around a centre in the new orthogonal space and are located in different quadrants. The inverse correlation between Cu, Zn and Sn and Pb indicates that samples with high amounts of Cu and Zn in the solid solution also have low amounts of Sn and Pb. Other elements, mainly As, Fe and Sb, are often present as traces (less than 0.3 wt.%) and do not correlate with Cu and Zn or Sn and Pb. This result suggests that their presence has little or no dependence on the concentration of alloying elements. They are generally associated with the copper ore; medieval ore processing is not comparable to modern techniques and many elements present in the copper ore still remain in the copper (albeit at lower levels).

**Fig 8:**
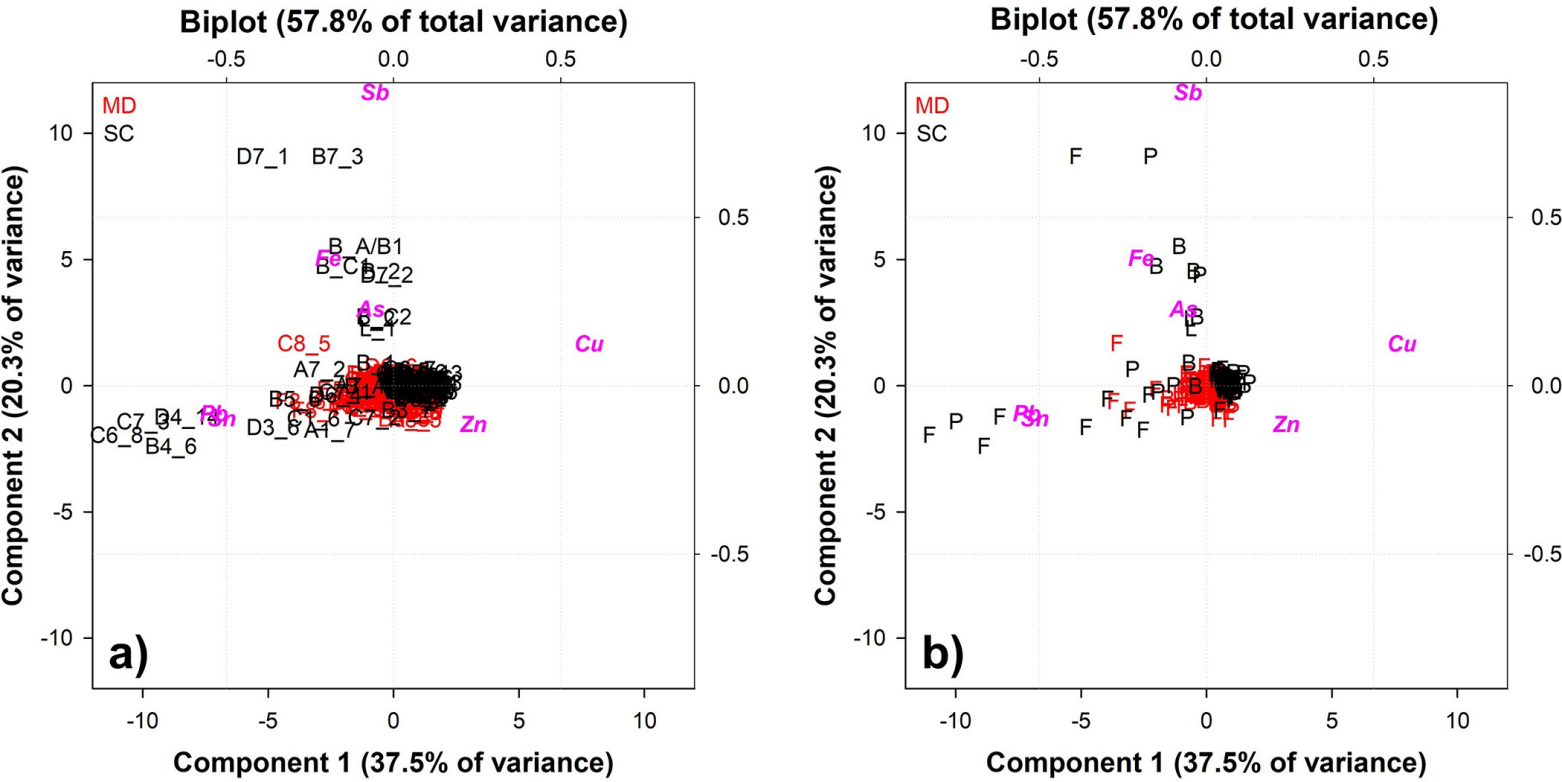
Biplot of the ED-XRF measurements of both Venice doors: MD = Main door in red; SC = San Clemente in black. **a)** ED-XRF number of measurements (**S2 Table**); **b)** coded samples: F = frames; P = plates; L = lionheads; B = spiral bars.

The biplot (loadings and scores) shows the alloy differentiation even more clearly (**Fig 8**). Two compositional macro-clusters are highlighted, corresponding to the two doors (MD in red; SC in black). The alloy used to produce the SC door is generally richer in Zn, while the MD has higher percentages of Sn and Pb (**Fig 8A**). There are also some outliers in the score plot, particularly for some SC points that appear far from the ’cloud’: these are mostly measurements taken from frames, lion heads and spiral bars, as can be seen from (**Fig 8B**). The points from the frames were considered as anomalies, possibly related to: i) the presence of the decorative inlays that influence the measurement (D7_1, B7_3, D7_2); ii) heterogeneities due to the corrosion products that do not give reliable results (B4_6, C6_8, C7_3, D4_14). According to [6], corrosion products were found on both doors: organic substances, oxides, sulphates, hydrochlorides, carbonates. These incrustations, heterogeneously distributed on the surface of the SC door, were detected by ED-XRF and must therefore be excluded. However, the analysis points obtained on the decorative elements (lion heads and spiral bars) do not fall into this category, as they appear to follow a specific pattern and require further study.

**Fig 9:**
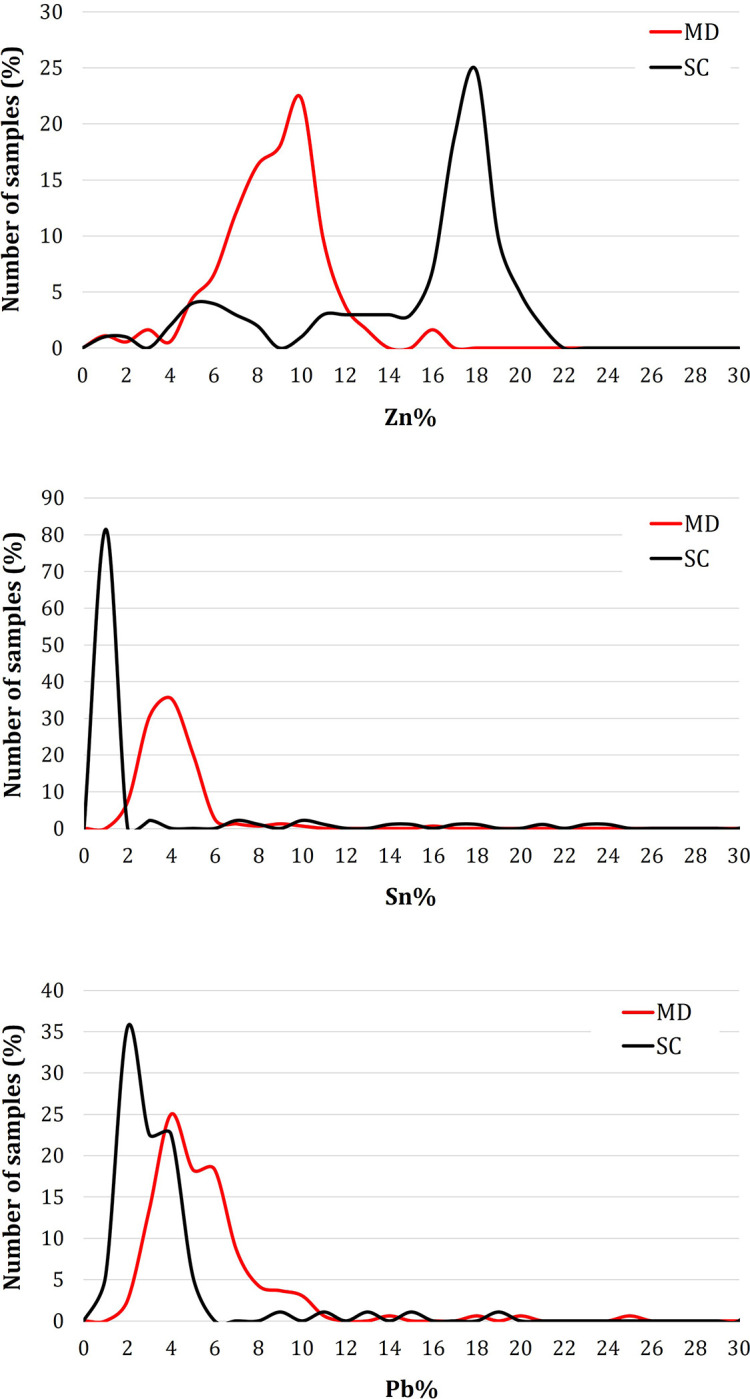
Frequency distribution of the main alloying elements detected in frames, plates and decorative elements in MD (red line) and SC (black line).

From the frequency distribution of the individual alloying elements (Zn, Sn and Pb; **Fig 9**) detected on MD (red line) and SC (black line), the major differences appear to be in the Zn and Sn contents rather than Pb.

Zn is much higher in SC, as confirmed by the PCA results, with an average of 18–19 wt.%, while MD shows lower averages, around 10 wt.%. As the Zn content increases, lower amounts of Sn are registered, less pronounced in SC than in MD (around 1 wt.% and 4 wt.% on average, respectively). A small overlap in the frequency distribution of Pb is also observed, indicating that the amount of Pb was similar for both doors (3 wt.% on average for MD and 5 wt.% for SC) and sufficient to confer good castability properties (i.e. pouring temperature, fluidity, etc…). These results strongly support the use of two different alloys: the alloy produced for the MD is quaternary (CuZnSnPb), while the SC door is made of leaded brass (CuZnPb).

**Fig 10:**
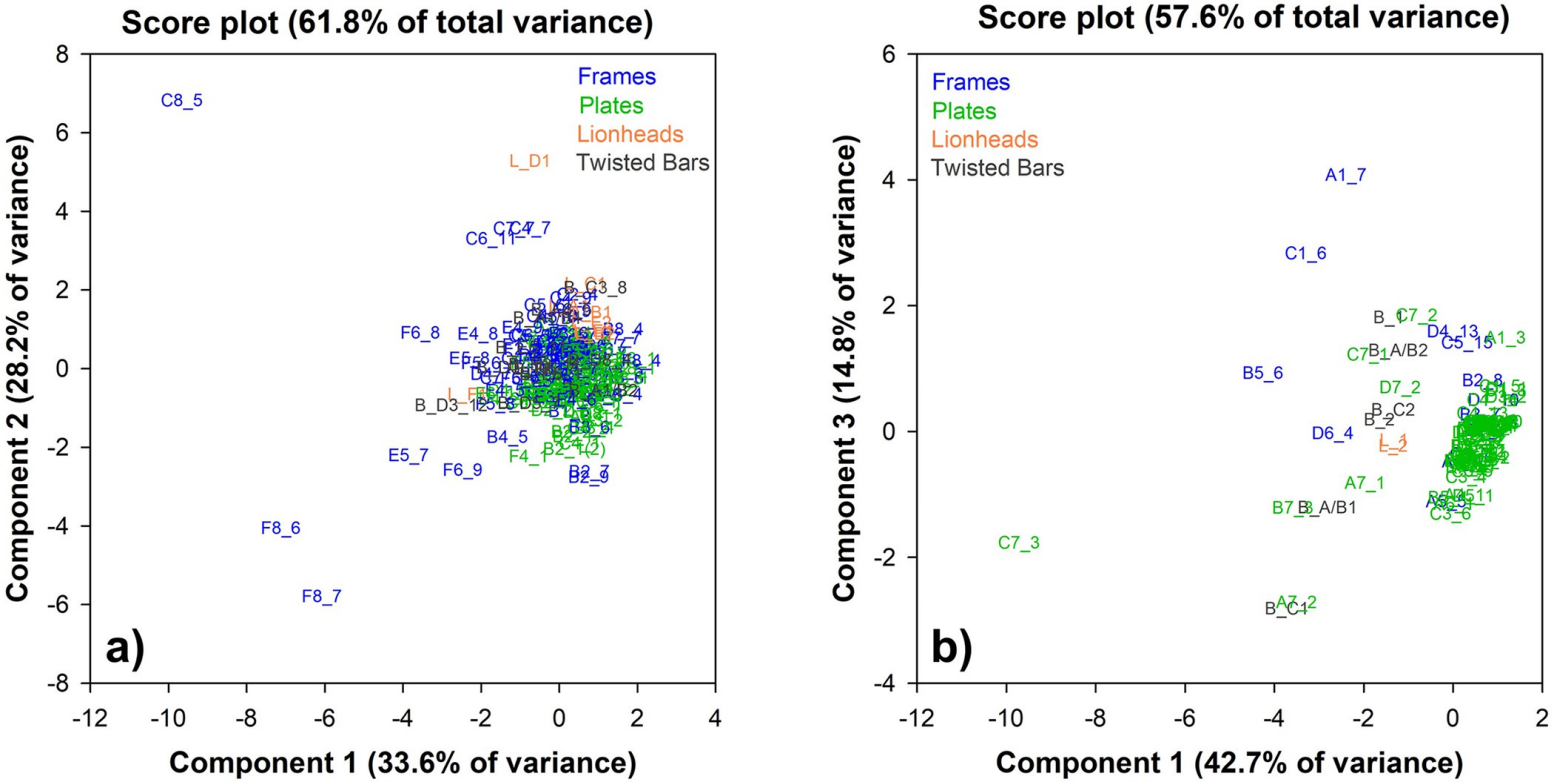
Score plot of the ED-XRF measurements on frames and plates of **a)** MD and **b)** SC. Coded samples (**S2 Table**) are distinguished by colour: frames = blue; plates = green; lionheads = orange; spiral bars = dark grey.

A PCA calculation was also carried out on the individual doors in order to distinguish possible clusters related to the area of measurement, namely frames, plates or decorative elements (lion heads and spiral rods). In this way, further information can be obtained to assess whether individual plates, frames or decorative elements have a significantly different chemical composition, indicating: i) a substitution of original plates; ii) and/or a different workshop/material. **Fig 10** shows the score plot of the two sets of MD and SC ED-XRF analyses.

**Fig 11:**
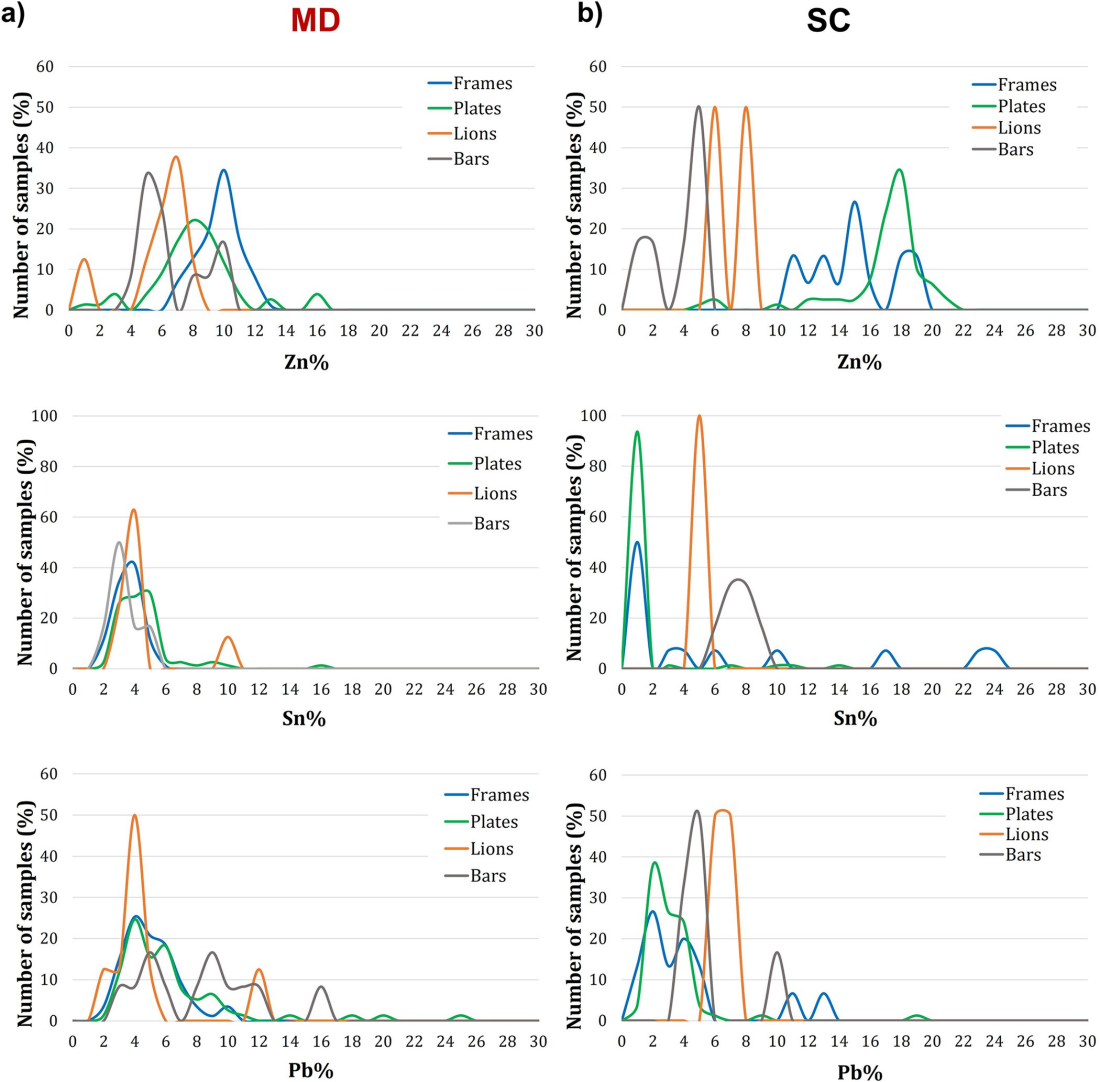
Frequency distribution of the main alloying elements detected in frames(blue), plates (green) lionheads (orange) and spiral bars (dark grey) of: **a)** MD; **b)** SC.

**Fig 10A**, a score plot of Component 1 vs Component 2 (61.8% of total variance), shows that MD is made from a quaternary bronze alloy: Plates, decorative elements and most of the frames have a similar chemical composition, as shown by the ’cloud’ of points concentrated between the I and II quadrants. Only the plates show a slight chemical differentiation: they are distributed in the upper part of the II quadrant, corresponding to a higher Zn content **(S1 Fig)**. The variation in the alloying elements is not as pronounced, as can be seen from the frequency distribution plot (**Fig 11**), with only small average compositional changes in Zn (8 wt.% for the plates and 10 wt.% for the frames).

Lionheads and spiral bars fall within the compositional range of frames and plates, suggesting the use of the same alloy for the entire door, with some points showing a lower Zn content, possibly due to a thicker surface layer of corrosion products. Some measurements on the frames (C8_5, F8_6 and F8_7), far from the cloud (**S2 Fig**), suggest the use of an alloy richer in Sn (average 8.2 wt.%), Pb (average 17.2 wt.%) and Fe (average 1.7 wt.%). These points were collected on the base under the door and may be related to the replacement of the original lower part with a modern alloy. They are also well distinguished in the frequency plot as small peaks in the Sn and Pb wt.% graphs.

The SC score plot (Component 1 vs Component 3, 57.6% of total variance—**Fig 10B**) shows a heterogeneous distribution of samples with different chemical compositions. The plates are concentrated in the II quadrant where the cloud of points shows an alloy rich in Zn (18 wt.%) and poor in Sn and Pb, confirming the use of a leaded brass. **Fig 11B** indicates that frames and plates have almost the same composition, with frames made of an alloy poorer in Zn (between 10 and 16 wt.%) and balanced by Sn and Pb. Some points of the frames (A1_7, B5_6, C1_6, D6_4) are considered as outliers due to heterogeneities of the corroded layers and are therefore excluded. Some measurements on the plates, namely the lower row of the door (A7 to D7), appear far from the plate cluster and towards a higher Sn and Pb content. Their composition reflects that of a quaternary bronze, as shown in **Fig 8B** and **S2 Table**. This trend is also observed for the lion heads and spiral bars (**S2 Fig**). Their composition is also very similar or mostly overlapping with that of the decorative elements produced for the MD, possibly suggesting the use of the same alloy for their manufacture.

**Fig 12:**
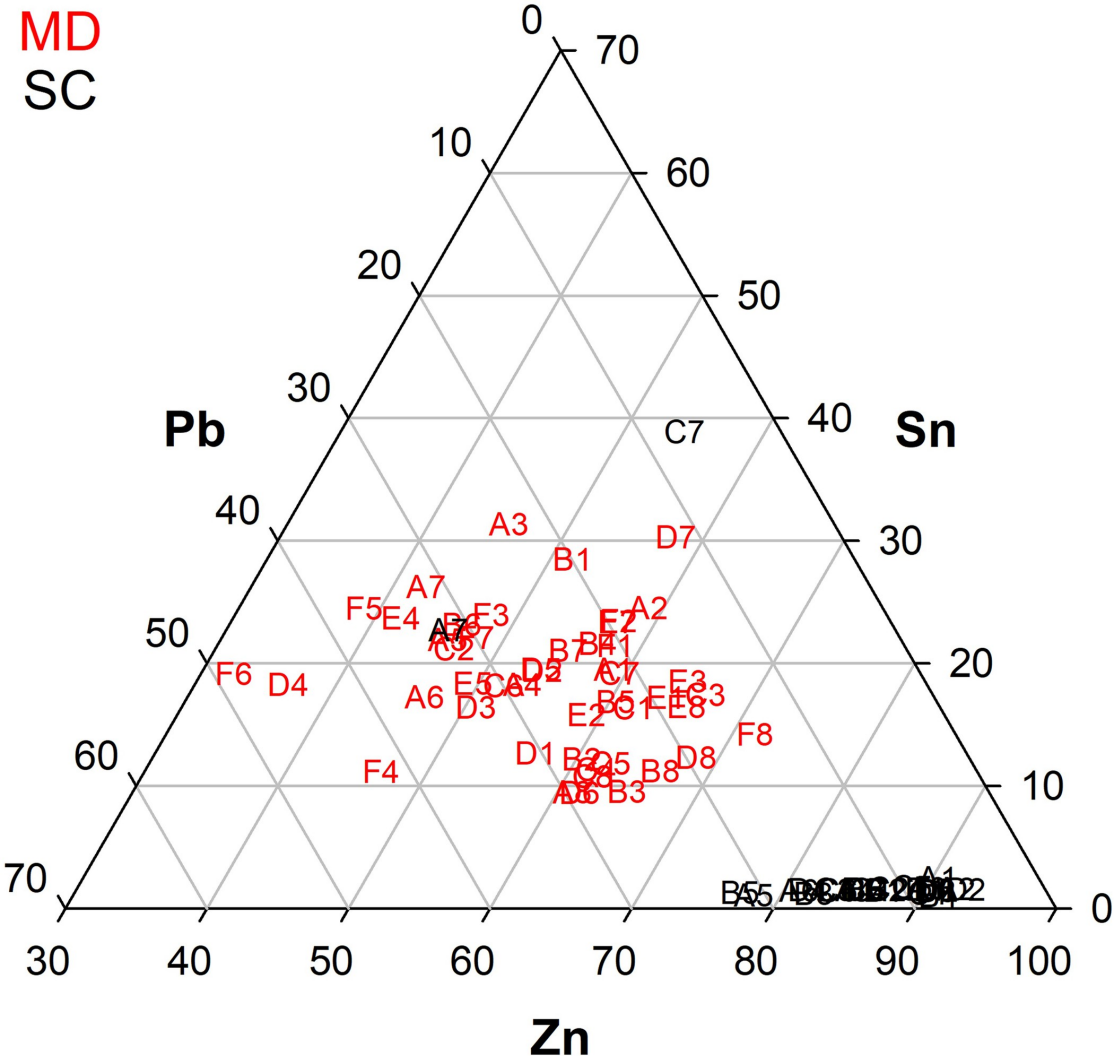
Ternary diagram of Zn-Sn-Pb of the plates average for MD (red) and SC (black).

In order to understand whether the plates of both doors had been replaced over the centuries, or whether some of them had been added later, further analysis was carried out. Plotting the average of the main alloying elements Zn, Sn and Pb on a reduced ternary diagram, two distinct clusters can be seen (**Fig 12**), confirming that two different types of alloy were used: quaternary for MD and leaded brass for SC.

This diagram also highlights the similarity of the composition of plates A7 and C7 of SC to the average composition of the MD alloy, suggesting a possible correlation between the plates. This result is consistent with the different decorative motifs that make up the lower part of the door (plates from A7 to D7).

#### 3.2.3 Manufacturing and provenance

The medieval bronze doors of the 11th and 12th centuries were usually made from a variety of copper alloys, all of which contained significant amounts of Pb, up to 15% by weight [1; and yet unpublished analyses by the authors]. Pb—an alloying element much cheaper than Sn or Zn—facilitated the casting process by increasing the fluidity of the alloy. As the cast plates did not undergo any significant deformation, but only a surface refinement, the high levels of Pb did not cause any problems in the further production of the bronzes. All of the metal plates of the nearly 30 doors that survive today were made of ternary or quaternary alloys: leaded tin-bronze, leaded brass, or Cu-Sn-Zn-Pb alloys. No specific alloy composition could be identified for the seven or eight bronze doors from Byzantium. Similarly, no specific alloy composition could be identified for the doors made entirely of copper alloys, i.e. not mounted on a wooden base (Hildesheim, Mainz, Palermo (Capella Palatina), Canosa, Rome (Battistero Lateranense)).

Comparing the chemical composition of the seven or eight byzantine doors, i.e. the doors imported from Byzantium to Italy (Amalfi, Atrani, Montecassino, Monte Sant’Angelo, Rome (San Paolo fuori le Mura), Salerno and Venice San Clemente, and, eventually, also the main door), we note that their plates are made either of leaded brass (Amalfi, Rome, Monte Sant’Angelo), leaded brass with up to 5 wt.% Sn (Atrani, Salerno, Venice San Clemente) or leaded tin bronze (the plates of Montecassino with Saints, surely deriving from Byzantium). Hence, even though we know they were all made in Byzantium, no uniform alloy composition can be noted.

Consequently, we can neither confirm nor exclude from the chemical composition of the plates and frames alone a local production of the main door from San Marco, Venice. The doors might also have been produced locally or be a later order from Byzantium. However, notably the metal parts below the lowest row of plates are likely of a more modern origin due to their high amount of Fe in the alloy. All other elements of the main door, that is: plates, frames, lion heads and spiral bars, are made of a quarternary CuSnZnPb alloy; variations in the object groups are mainly related to the surface corrosion (especially in the lower parts of the door) and segregation during cooling.

The door of San Clemente instead was made of leaded brass (plates and frames); heterogeneity in the Zn-amount of the plates is related to corrosion. The lowest row of the plates, the lion heads and the spiral bars instead are made, as all components of the main door, of a quaternary CuSnZnPb alloy. This could be explained by eventually later works on the door, eventually even during the production of the main door, in order to create a basic, stylistic similarity between the doors (lion heads and spiral bars). However, the stylistic similarity of the plates in the last row with the decoration of the frames and the other plates is striking, assuming the production in the same workshop or by the same artist.

### 3.3 Wood analyses

**Fig 13:**
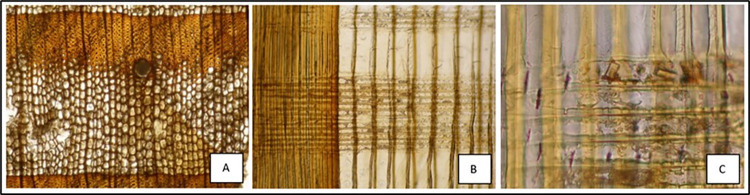
Microscopic features of the woods from San Clemente’s door. A and B: cross and radial section of larch. C: radial section of silver fir.

Microscopic analysis showed that most of the structural wood in both doors is larch (*Larix decidua* Mill., **Fig 13**). Among our samples, only those from the inner layer next to the bronzes of the San Clemente door turned out to be made of another wood species, namely silver fir (*Abies alba* Mill.) (**Table 1**). In the case of the main door, there is no way of verifying the presence of a corresponding layer of wood, since all the sides of both leaves are completely covered with metal elements.

**Fig 14:**
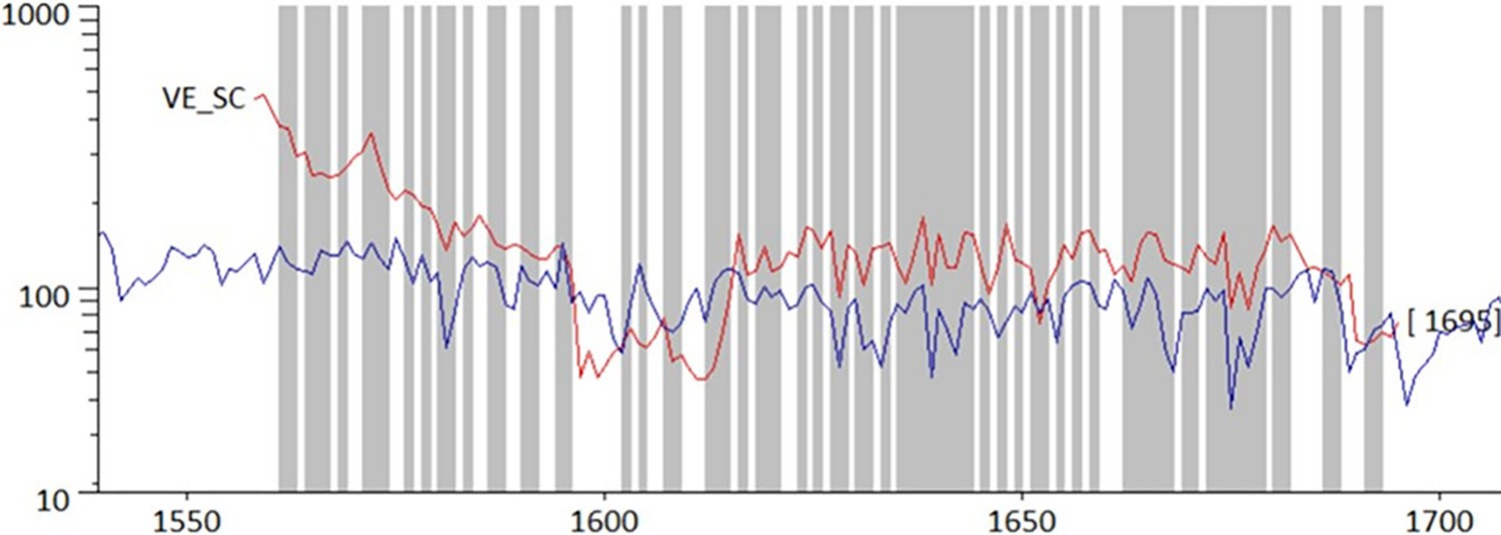
Visual cross-matching of the San Clemente’s larch chronology (VE_SC, in red) and Bernabei-Bontadi’s reference chronology (2018). The grey areas display the periods of common *Gleichlauf*, a measure of the year-to-year agreement.

The tree-ring sequences from the pictures taken on the vertical boards of the San Clemente Door cross-date very well with each other and have therefore been grouped into the mean chronology VE_SC, which spans 138 years (**Fig 14; Table 2**). Comparing the San Clemente door larch chronology with a number of reference chronologies, the best correlations were obtained with high-altitude chronologies from the Eastern Alps, i.e. our own Tonale larch chronology [23]. The last year of the larch wood of the San Clemente door is 1695 CE. Since they are planks, the dated wooden elements were trimmed during their production and therefore do not show any traces of the waney edge, i.e. the last ring, close to the bark, produced by the living tree before felling. In addition, the processing of the boards removed all sapwood, the outer part of the trunk recognizable in larch by its lighter colour, made up of a more easily degradable wood. In view of the above, the date of 1695 CE must be considered a *post quem* date, a threshold of time before which the wooden structure of the San Clemente door could not have existed. Mature larch trees usually have an average of 30–50 growth rings in the sapwood: this means that at least 30 years can be added to the date of 1695 CE to obtain an estimate of the most likely date of felling. Also taking into account the time needed for harvesting, transport and seasoning, it is reasonable to assume that the wooden structure of the San Clemente Gate was certainly built after 1730–1740 CE.

**Table 2:** Cross-dating results of the San Clemente’s larch chronology (VE_SC) for the year 1695 with reference chronologies. T_BP_ and T_HO_: t-value adapted to time-series by [25, 26]; Glk, *Gleichläufigkeit*, represents the percentage of the agreement between the sign of the growth from one year to another [27]; *, ** and ***, significance level of the correlation coefficient, assessed respectively at p = 0.05, 0.01 and 0.001.

Region/Site	Reference	Overlap	T_BP_	T_HO_	Glk (%)
Val di sole (TN, Italy)	[24]	138	4.51	5.17	57.80*
Eastern Italian Alps orientali italiane	[27]	138	4.44	4.88	58.00*
Malta (structural timber)	[28]	103	4.63	4.62	61.90**

In the case of the main door, the presence of a thick layer of paint over the entire wooden structure and the presence of metal elements on the sides, which hide all the cross sections of the boards, made dendrochronological analysis inapplicable. However, the similarity of the two wooden structures and the presence of the same type of boards for both doors make it reasonable to assume that they were made together at the same time.

## 4. Conclusion

Combining the results of the chemical analyses, it is very likely that the main door was also made at a later date in Constantinople. While the first door (San Clemente) in Venice was probably made in an Amalfitan workshop in Constantinople in the early 1080s, the conditions under which the second door was made must have been somewhat different. During the 12^th^ century, Pisa and Venice dominated maritime trade with Constantinople, while Amalfi became increasingly insignificant [29]. It is therefore possible that the second door for the Basilica di San Marco was made in another workshop in the city on the Bosporus, which nevertheless benefited from the technical knowledge of the older Amalfi workshops.

This second workshop probably also made the lion heads and spiral borders on the SC door, as indicated by the similar alloys used. This was probably done to make the two doors more aesthetically uniform. Closer inspection reveals that the lion heads on the SC door cover some of the filigree frame ornamentation, which would not have been the case with the door pulls that were originally planned. Furthermore, these later additions to the SC door at the same time as the production of the MD suggest a close exchange between the Venetian patrons and the workshops and artists in Constantinople. This is also suggested by the inclusion of many Venetian saints in the depictions on the MD.

It remains to be seen why the bottom four plates of the PC door are also made from this alloy. Their ornamentation corresponds in technique, design and colouring to the frame system of the same door, suggesting a contemporary origin. It is possible, for example, that more material was added during the production process to cast the last row, because the prepared material was insufficient, or something similar.

In more recent time, toward the end of the first half of the 18th century or later, the doors underwent restoration work that involved the renovation of the wooden structure and the rearrangement of the plates (originally the saints were probably facing the centre of the Basilica di San Marco). Today, both doors have a multi-layered structure in larch, which, at least in the case of the San Clemente door, covers a third layer in silver fir.

## Supporting information

S1 Fig Frames elements brazed together. Left: plate F6; right plate F7.
(TIF)

S2 Fig Loading plot of the ED-XRF measurements on frames, plates and decorative elements of MD and SC. (TIF)

S1 Table Standard alloys used for calibration. SEM-EDXS analyses performed on intentionally produced copper alloy samples confirmed the ED-XRF accuracy.
(XLSX)

S2 Table Chemical composition of both doors. (XLSX)
