# Supplementary figures and images for: Multidisciplinary analyses on the 11th-12th century bronze doors of San Marco, Venice
Source: PLoS One. 2023 Jul 13;18(7):e0288094. doi: 10.1371/journal.pone.0288094 (PMC10343026; doi:10.1371/journal.pone.0288094)

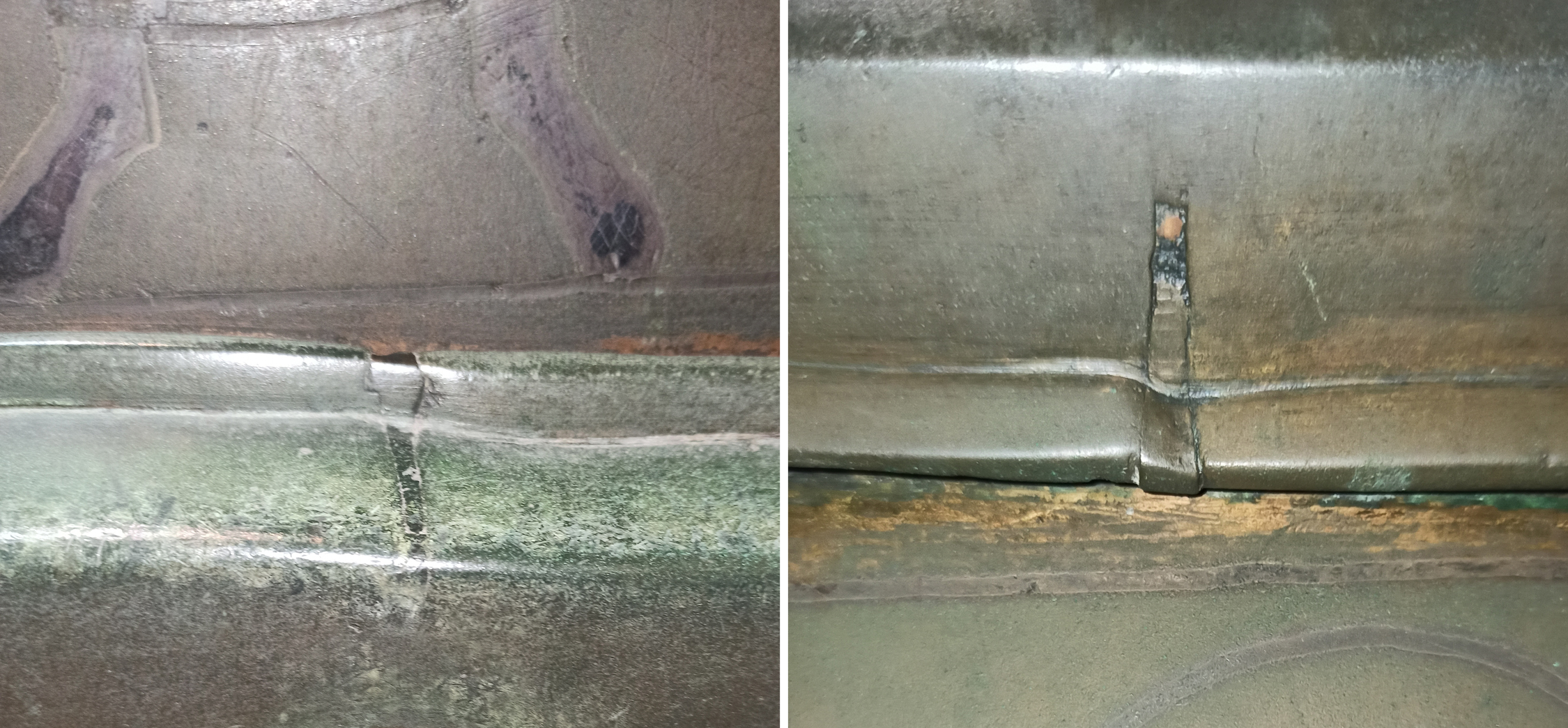

Supplement: S1 Fig — Left: plate F6; right plate F7. (TIF) [file pone.0288094.s001.tif]

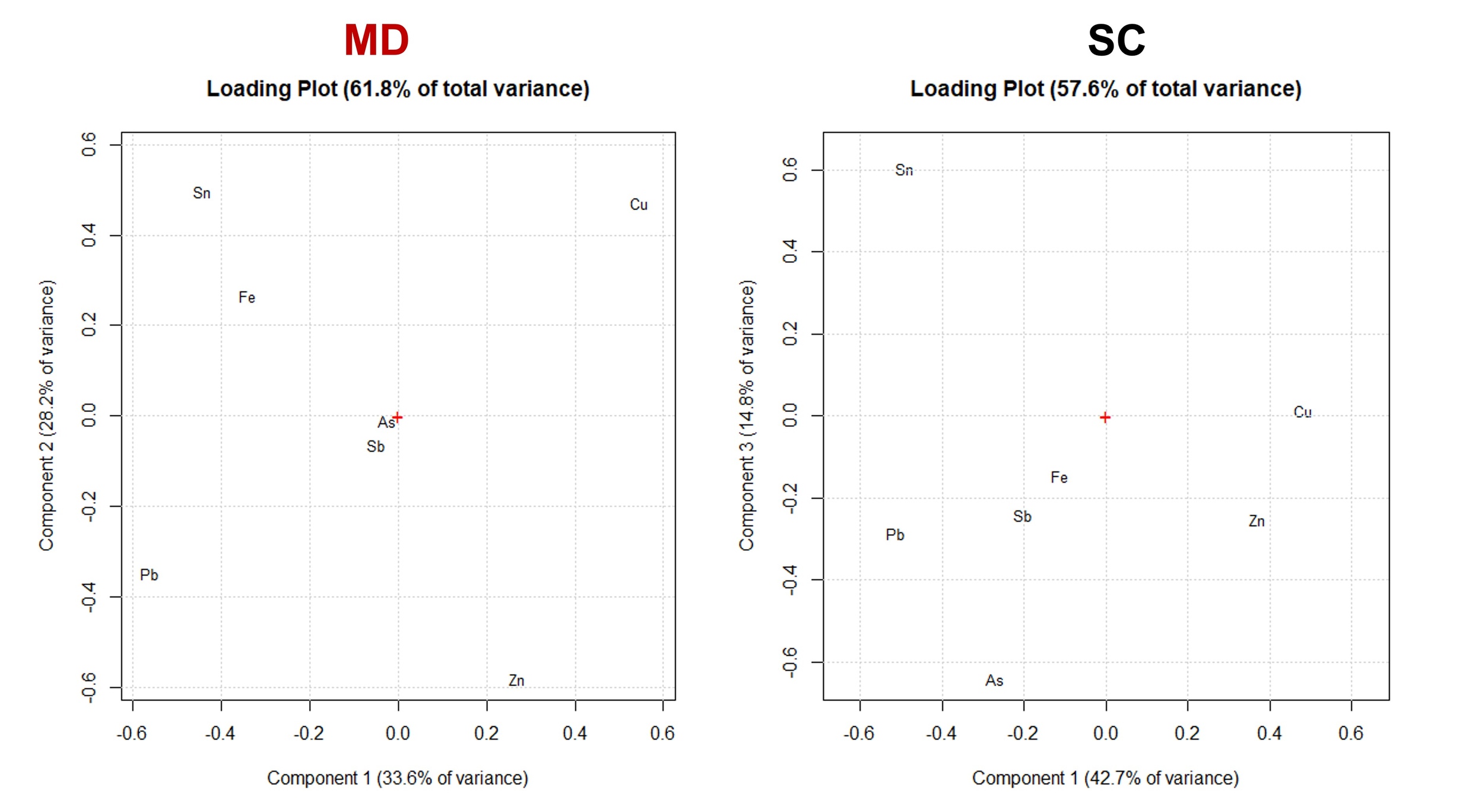

Supplement: S2 Fig — (TIF) [file pone.0288094.s002.tif]
